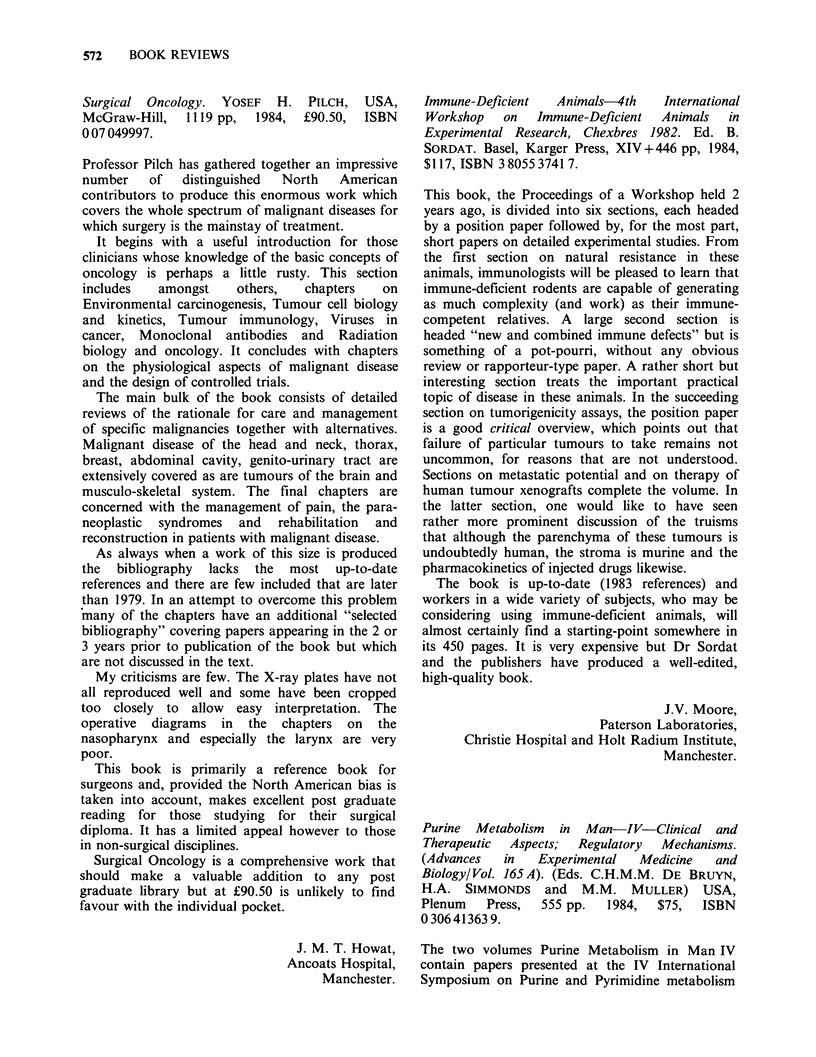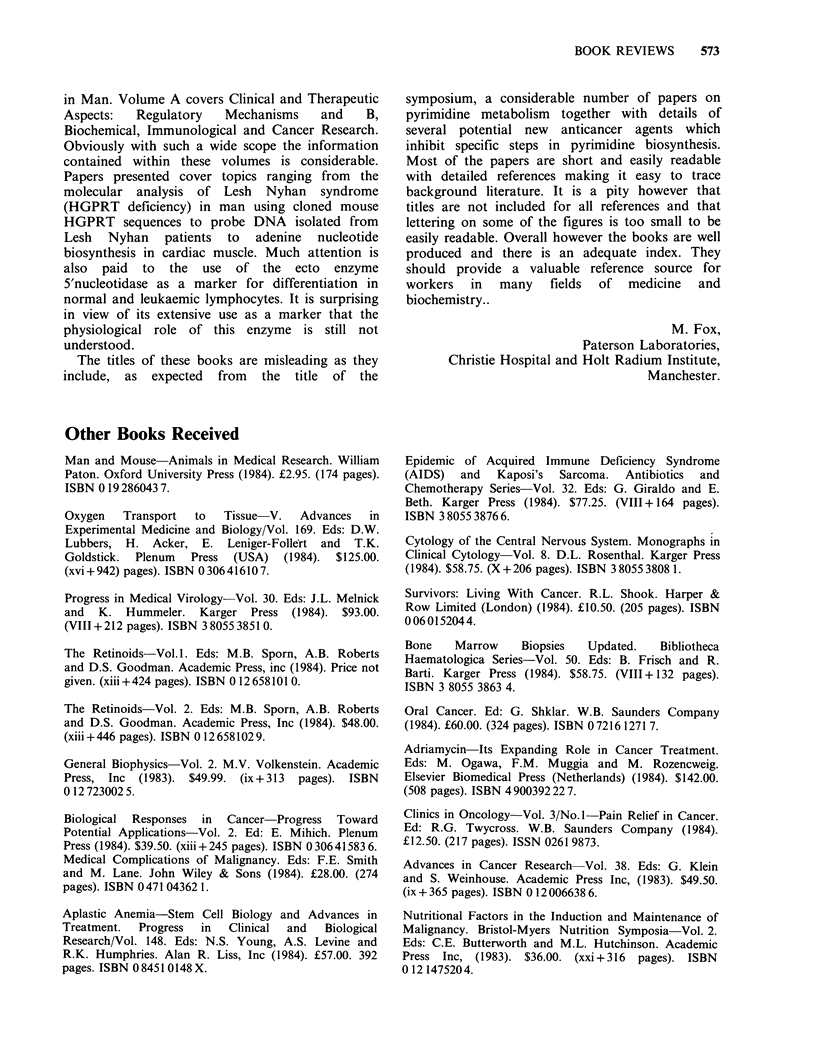# Purine Metabolism in Man—IV—Clinical and Therapeutic Aspects; Regulatory Mechanisms. (Advances in Experimental Medicine and Biology/Vol. 165 A)

**Published:** 1984-10

**Authors:** M. Fox


*Purine Metabolism in Man—IV—Clinical and Therapeutic Aspects; Regulatory Mechanisms. (Advances in Experimental Medicine and Biology/Vol. 165 A)*. (Eds. C.H.M.M. DE BRUYN, H.A. SIMMONDS and M.M. MULLER) USA, Plenum Press, 555 pp. 1984, $75, ISBN 0 306 41363 9.

The two volumes Purine Metabolism in Man IV contain papers presented at the IV International Symposium on Purine and Pyrimidine metabolism in Man. Volume A covers Clinical and Therapeutic Aspects: Regulatory Mechanisms and B, Biochemical, Immunological and Cancer Research. Obviously with such a wide scope the information contained within these volumes is considerable. Papers presented cover topics ranging from the molecular analysis of Lesh Nyhan syndrome (HGPRT deficiency) in man using cloned mouse HGPRT sequences to probe DNA isolated from Lesh Nyhan patients to adenine nucleotide biosynthesis in cardiac muscle. Much attention is also paid to the use of the ecto enzyme 5′nucleotidase as a marker for differentiation in normal and leukaemic lymphocytes. It is surprising in view of its extensive use as a marker that the physiological role of this enzyme is still not understood.

The titles of these books are misleading as they include, as expected from the title of the symposium, a considerable number of papers on pyrimidine metabolism together with details of several potential new anticancer agents which inhibit specific steps in pyrimidine biosynthesis. Most of the papers are short and easily readable with detailed references making it easy to trace background literature. It is a pity however that titles are not included for all references and that lettering on some of the figures is too small to be easily readable. Overall however the books are well produced and there is an adequate index. They should provide a valuable reference source for workers in many fields of medicine and biochemistry..

M. Fox,
Paterson Laboratories,
Christie Hospital and Holt Radium Institute,
Manchester.